# Finite element analysis comparison of Type 42A2 fracture fixed with external titanium alloy locking plate and traditional external fixation frame

**DOI:** 10.1186/s13018-023-04307-1

**Published:** 2023-10-31

**Authors:** Shitao Fang, Ling Zhang, Yunqi Yang, Yun Wang, Jinkun Guo, Lei Mi

**Affiliations:** 1 Department of Orthopaedic Medicine Center, Brain Hospital of Hunan Provincial (The Second People’s Hospital of Hunan Province), Clinical Medical College of Hunan University of Chinese Medicine, Changsha, 410007 China; 2 Department of Nursing, The Third Xiangya Hospital of Central South University, Changsha, 410013 China

**Keywords:** Finite element, Tibial fracture, External titanium alloy locking plate, External fixation bracket, Biomechanics

## Abstract

**Background:**

At present, not all Type AO/OTA 42A2 open fractures can be treated by external fixation brackets, not to mention the inconvenience of this technique in clinical practice. External titanium alloy locking plates, which are lightweight and easy-to-operate, can be used as an alternative treatment option for such patients. However, there are few reports of finite element biomechanical analysis on the titanium alloy locking plates and fixation brackets being placed on the medial side of the tibial fracture. In this study, the biomechanical properties of titanium alloy locking plates and fixation brackets for treating Type AO/OTA 42A2 fractures were compared by applying the finite element method, and the results provided data support for the clinical application of the external titanium alloy locking plate technique.

**Methods:**

Type AO/OTA 42A2 fracture models were constructed using CT data of a male volunteer for two external fixation techniques, namely the external titanium alloy locking plate technique and the external fixation bracket technique, according to commonly-used clinical protocols. Then, the four-point bending, axial compression, clockwise rotation and counterclockwise rotation tests under the maximum load were simulated in finite element analysis software. The stress distribution, peak stress and overall tibial displacement data for the two different external fixation techniques were obtained and compared.

**Results:**

In the four different test conditions (i.e., four-point bending, axial compression, clockwise torsion, counterclockwise torsion) under the maximum load, the two external fixation techniques showed obvious von Mises stress concentration at the contacts between the screw and tibia, between the screw and titanium alloy locking plate, between the self-tapping self-drilling needle and tibia, between the self-tapping self-drilling needle and the external fixation device, as well as around the fracture end and around the cortical bone at the upper and lower ends of the tibia. The peak stress was ranged 26.67–558.77 MPa, all below the yield stress strength of titanium alloy. The peak tibial displacement of the external titanium alloy locking plate model was smaller than that of the fixation bracket model. In terms of structural stability, the external titanium alloy locking plate technique was superior to the external fixation bracket technique.

**Conclusions:**

When fixing Type AO/OTA 42A2 fractures, external titanium alloy locking plates are not only lightweight and easy-to-operate, but also have better performance in terms of axial compression, bending and torsion resistance. According to the finite element biomechanical analysis, external titanium alloy locking plates are superior to traditional external fixation brackets in treating Type AO/OTA 42A2 fractures and can better meet the needs of clinical application.

## Introduction

Tibial fractures, as a common type of long bone injury, are often open fractures caused by violent factors [1, 2]. The healing of such fractures is usually more difficult due to susceptibility to infection, injury of skin and soft tissues, and insufficient blood supply to the mid-tibia [3]. Generally speaking, for the treatment of open fractures, external fixation is more suitable than internal fixation [4, 5], especially when the patients involve severe skin and soft tissue injuries [6]. The traditional external fixation brackets placed on lower limbs are heavy and bulky, therefore prone to causing mobility difficulties and discomfort to the patients’ daily life and appearance [7]. Particularly, for some special patients, such as those with psychiatric disorders, there is even a risk that the patient may remove the bracket before the fracture reaches clinical healing due to a low degree of cooperation [8, 9]. In comparison, external titanium alloy locking plates are lightweight and comfortable and have little impact on the patients’ dressing and walking [10]. Meanwhile, external titanium alloy locking plates are characterized by low notching, good stability, minor skin irritation, and low possibility of infection [11], and have demonstrated excellent efficacy in the clinical treatment of tibial fractures [12–14]. When titanium alloy locking plates are properly fixed, the bone plate, screws and tibia will form a structure with high axial stability [15]. However, the tibia often lacks a fixed tension side. To date, there are few reports regarding where the fixation should be placed [16], and the feasibility of external titanium alloy locking plates has not been widely recognized. Therefore, in this study, we constructed a finite element model by empirically placing the external titanium alloy locking plate on the medial side of the tibia to simulate its performance in the human body under different surgical conditions. Then, the relevant performance indicators were analyzed for the purpose of providing clinical reference. Subsequently, models of the external titanium alloy locking plate technique and the traditional fixation bracket technique for the treatment of Type AO/OTA 42A2 fractures were established and compared in terms of structural stability and clinical feasibility. We expected to provide theoretical support and data reference for clinicians.

## Materials and methods

### Research subjects

The tibial computed tomography (CT) scan data (0.625 mm thickness, Digital Imaging and Communications in Medicine (DICOM) format) of a male volunteer (25 years old, height 173 cm, weight 69.5 kg) who underwent X-ray examinations of the tibia in anteroposterior and lateral, dual-oblique and dynamic positions at our hospital were acquired to reconstruct the three-dimensional (3D) tibial model. The patient had been ruled out of tibial deformity, fracture, tumor, infection and other diseases, and had signed informed consent to participate in this study. This study was approved by the Medical Ethics Committee of Hunan Brain Hospital.

### Equipment and software

The equipment and software used in this study include: 64-row spiral CT machine (voltage, 120 kV; current, 100 mA; layer thickness, 0.625 mm), Mimics 21.0 software (Materialise Company, Belgium), Geomagic Studio 2014 software (Raindrop Company, America), Hypermesh 14.0 software (Altair Company, America), MSC.Patran 2019 software (Hexagon Company, Sweden), and MSC.Nastran 2019 software (Hexagon Company, Sweden).

### Methods

Mimics 21.0 (Materialise Company, Belgium) was used to extract the desired tibial CT data and to reconstruct the normal complete stereolithography (STL) tibial model. In Geomagic Studio 2014, the CT data were repaired, de-noised and surfaced, and were reverse-processed to obtain the normal tibia. Then, two different external fixation techniques (i.e., external titanium alloy locking plates, and unilateral external fixation brackets) were simulated for the treatment of Type AO/OTA 42A2 fractures and the corresponding STP models were established. The STP file was imported into Hypermesh 14.0 for meshing, and a Glyph Bitmap Distribution Format (BDF) file was exported for later-stage finite element mesh configuration, parameter configuration, load application, boundary constraint configuration, and various calculations in MSC.Patran 2019 and MSC.Nastran 2019.

#### Image acquisition

With the volunteer placed in a supine position, a 64-row spiral CT machine was used to scan both lower limbs from the foot to the position 10cm above knee joint. A total of 598 CT images were obtained and saved in DICOM format.

#### Construction of the 3D tibial model

**Fig. 1:**
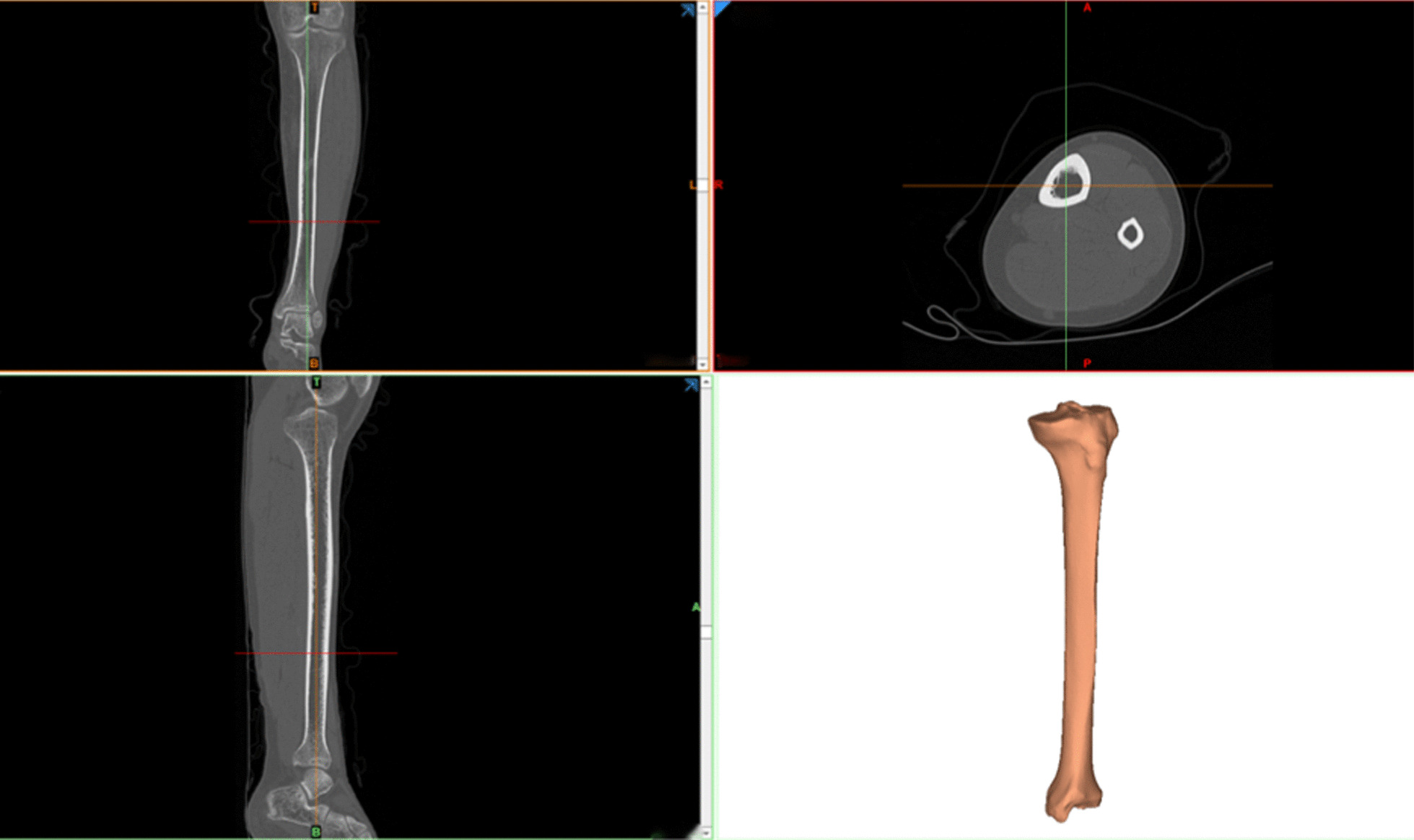
CT image data three-dimensional reconstruction of tibial diagram

- *Image input stage* The CT data in the DICOM file were imported into Mimics21.0 and were converted into a corresponding engineering file in order to further obtain the 3D image information of the tibial structure.
- *Threshold determination stage* The cross-sectional view of the tibia was obtained by scanning in different views (coronal, horizontal, sagittal), and the structural soft tissue shadow was removed properly. Then, by adjusting the contrast and grayscale of the scanned image, the default threshold of the skeleton system was selected to define the threshold of the tibial image. The HU value is 226 ~ 2048.
- *Image segmentation and filling, and generation of 3D model* We used editing tools to cover the shape of the image, select the tibial area, select the region growing, and perform relevant repair, erase and other commands to obtain the outer skeletal outline of the tibia. Finally, the “Calculate 3D” tool was used to generate the triangular geometric model.
- *Optimization of the 3D model* The “Remesh” module was used to triangulate the 3D tibia model, and the “STL Smoothener” tool was used to smooth the triangular surface mesh. The “Angle” tool was used to increase the parameter value of the angle, and the “Point” and “Edge” tools were used to adjust the number of triangles. Then, the “Reduced with quality” command was used to count the number of erroneous triangles and delete unqualified and overlapping triangular faces. Eventually, an accurate STL tibial model with a highly approximate geometric shape, a smooth surface and a high triangular mesh quality was obtained, as shown in Fig. 1.

##### Surface fitting and geometric solid model processing

The preliminary tibia 3D model generated in Mimics was imported into Geomagic Studio2014 (Raindrop Company, America) in STL file format to derive the tibia contour cloud map. After polishing, nail removal, noise removal and relaxation, a satisfactory shape of the tibia was obtained. The processed model was then examined using the grid Doctor function in Geomagic Studio in order to ensure that there were no redundant, defective, or irregular parts. Subsequently, the model was further optimized by detecting and editing the contour line, constructing and refining the surface sheet, constructing the grating, and finally fitting the surface. The optimized tibia model was then shifted inward by 2 mm using the offset function and was further processed by the precision surface function to generate the cancellous bone model of the tibia.

#### Construction of solid external fixation models

The created 3D models of the tibial cortical bone and tibial cancellous bone were further imported into Geomagic Studio 2014 to construct the overall tibia model using the assembly function. Then, the overall tibial model was reversely processed on the XY plane using the segmentation function. The fracture ends were treated as gapless and set to face-to-face contact state. Finally, the AO/OTA 42A2 fracture model was established.

**Fig. 2:**
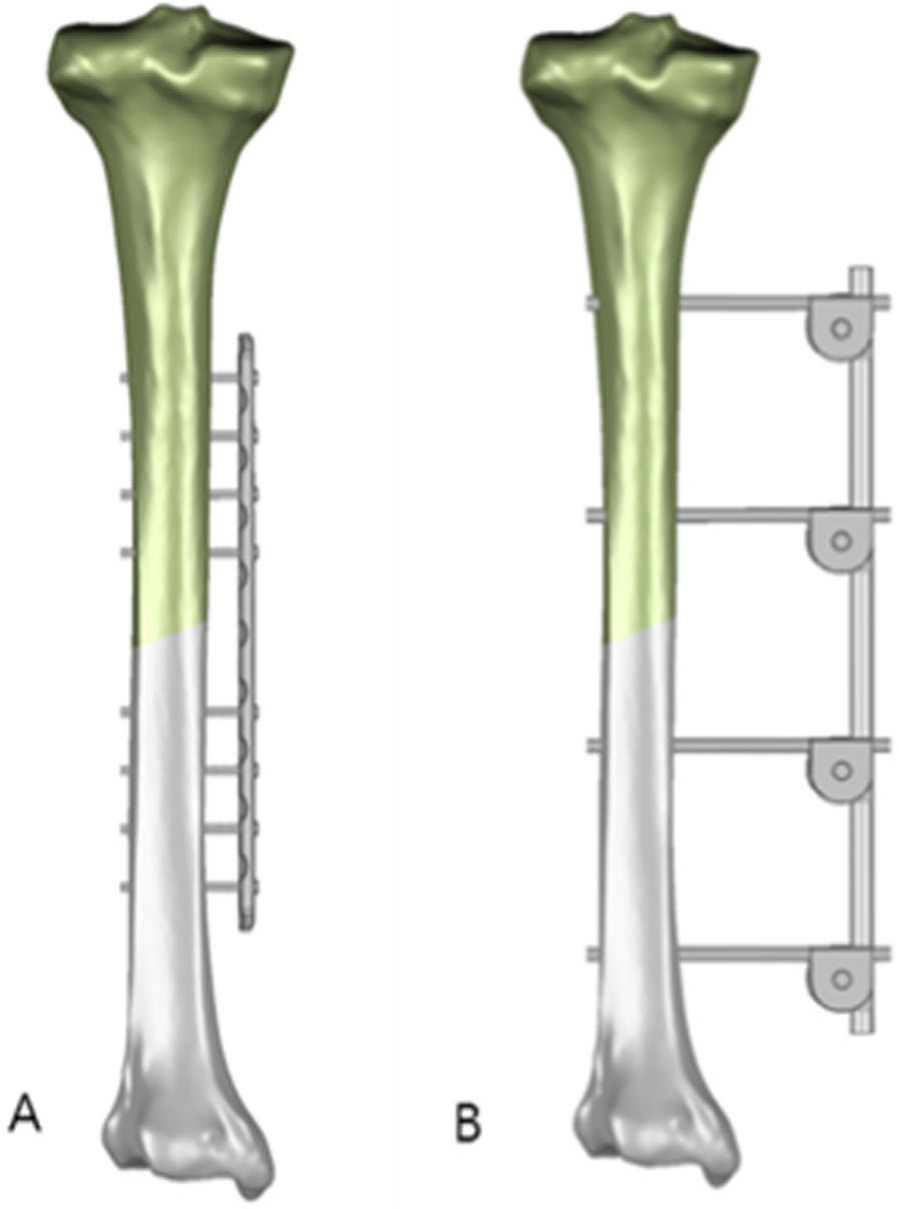
Reverse diagram of external titanium alloy locking plate and unilateral external fixation bracket fixed tibia. **A** External titanium alloy locking plate group; **B** external fixation bracket group

The next step was to prepare for the establishment and assembly of external fixation devices. Specifically, the titanium alloy locking plate (Titanium alloy locking plate, Double Medical Technology Inc, China) used in this study consisted of 10 holes, with overall dimensions of 191.0 mm long * 14.0 mm wide * 4.6 mm thick. The plate was placed in the inner side of the tibial fracture model, and four screws, with a diameter of 3.0 mm, were installed at the upper and lower ends of the fracture surface, respectively. The unilateral external fixation bracket (One-arm one-piece external fixator, Double Medical Technology Inc, China) had a length of 250.0 mm. Two self-tapping self-drilling needles, with a diameter of 5.0mm, were installed at the upper and lower ends of the fracture surface, respectively, which were connected by a rod with a diameter of 8.0mm. The design for the contact between the external fixation device and the bone adopted the most commonly-used principles and protocol in clinical practice. The standard rod length of the bracket for large bone fracture fixation is 11mm. However, in this study, the 8mm protocol, which is more widely used by clinicians, was applied, as this length provides better moment of inertia and stability and is expected to exhibit superior biological properties. Therefore, it might serve a better benchmark for the comparison with the titanium alloy locking plate technique. Eventually, the STP models for Type AO/OTA 42A2 fractures treated by the two different external fixation techniques (i.e., external titanium alloy locking plate, and unilateral external fixation bracket) were established, as shown in Fig. 2.

#### Finite element meshing

The STP files of Type AO/OTA 42A2 fractures fixed by two different external fixation techniques were imported into Hypermesh 14.0 for meshing. The exported BDF files were further imported into the finite element preprocessing software MSC.Patran 2019 for mesh configuration, material parameter configuration, load application, and boundary constraint configuration. Then, the finite element post-processing software MSC.Nastran 2019 was used for computational analysis. The tibial cortical bone, cancellous bone, and external fixation devices (external titanium alloy locking plate and unilateral external fixation bracket) for the two different techniques were all simulated by tetrahedral solid mesh elements (TetMesh Tet4 Element). The numbers of elements and nodes are shown in Table 1.

**Table 1:** Meshing of finite element groups

Sequence	Constituencies	Number of nodes (pcs)	Number of units (pcs)
1	External titanium alloy locking plate group	68,351	329,920
2	External fixation bracket group	55,968	276,825

#### Parameter configuration

Referring to the relevant data in the published literature [17–19], the material parameters in this study were configured as shown in Table 2. It was assumed that the tibial cortical bone, cancellous bone, and external fixation devices were all isotropic, uniform, and continuous linear elastic materials. According to the contact settings in previous studies [19, 20], the fracture surface was considered to be in a completely fractured state, with non-co-node contact (contact friction coefficient, 0.3). Moreover, in order to simulate the locking effect of titanium alloy plate screws, the contacts between the screw and titanium alloy plate or bone were set as co-node binding for the purpose of ensuring reliable and tight connection.

**Table 2:** Structural material parameters of tibia and external fixation

Name	Elastic modulus (MPa)	Poisson's ratio
Tibial cortical bone	17,000	0.30
Tibial cancellous bone	700	0.20
External fixation (Ti–6Al–4V)	106,000	0.33

#### Assumption of boundary conditions

The theoretical calculations in this study mainly aimed to reveal the stress condition in the linear elastic static stage, and the force and load basically exhibited a linear relationship. Therefore, referring to the relevant literature [21–24], the load was directly applied to the maximum level during the simulation process in all the four test conditions (four-point bending, axial compression, clockwise rotation, and counterclockwise rotation) in order to analyze the strength and stability of the tibia after oblique fractures treated by two different external fixation techniques. The four test conditions were implemented as follows: (1) Four-point bending test: Constraint fixation was implemented at the distal and proximal ends of the tibia, with the external force applied from the front to the back [25]. Then, a bending load of 300N was applied to both ends of the fracture gap [24]. (2) Axial compression test: Constraint fixation was implemented at the distal end of the tibia, and an axial compressive load of 600N was applied on the proximal tibia platform [21–23]. Meanwhile, in order to ensure consistent boundary conditions as biomechanical axial compression test, part of the nodes around the proximal tibia platform were constrained to limit lateral movement while only allowing vertical downward movement. (3) Clockwise rotation test: Constraint fixation was implemented at the distal end of the tibia, and a torque load of + 5N·m was applied to the proximal tibial platform [24]. (4) Counterclockwise rotation experiment: Constraint fixation was implemented at the distal end of the tibia, and a torque load of -5N·m was applied to the proximal tibial platform [24].

### Main observation indicators

By conducting finite element simulations on the two different external fixation techniques for Type AO/OTA 42A2 fractures, the von Mises equivalent stress cloud map and displacement cloud map of the tibia and the external fixation devices under the maximum load were obtained for all the four test conditions. The test data was used to compare the two fixation techniques in terms of stress distribution, peak stress, and overall tibial displacement in each test condition.

## Results

### Von Mises stress distribution and peak stress

**Fig. 3:**
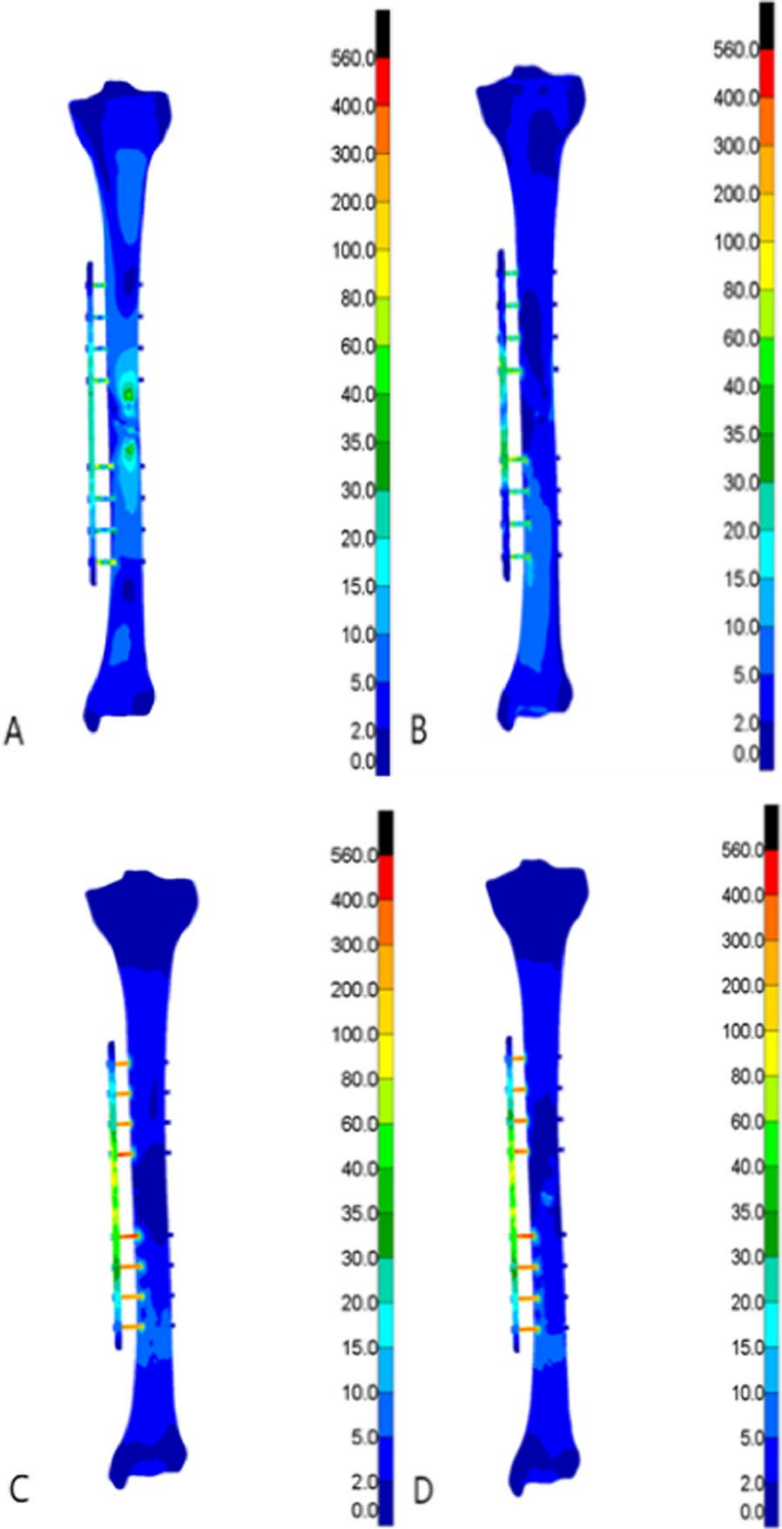
Von Mises stress cloud diagram under four experiments of external titanium alloy locking plate group (MPa). **A** Four-point bending experiment; **B** axial compression experiment; **C** clockwise torsion experiment; D counterclockwise torsion experiment

**Fig. 4:**
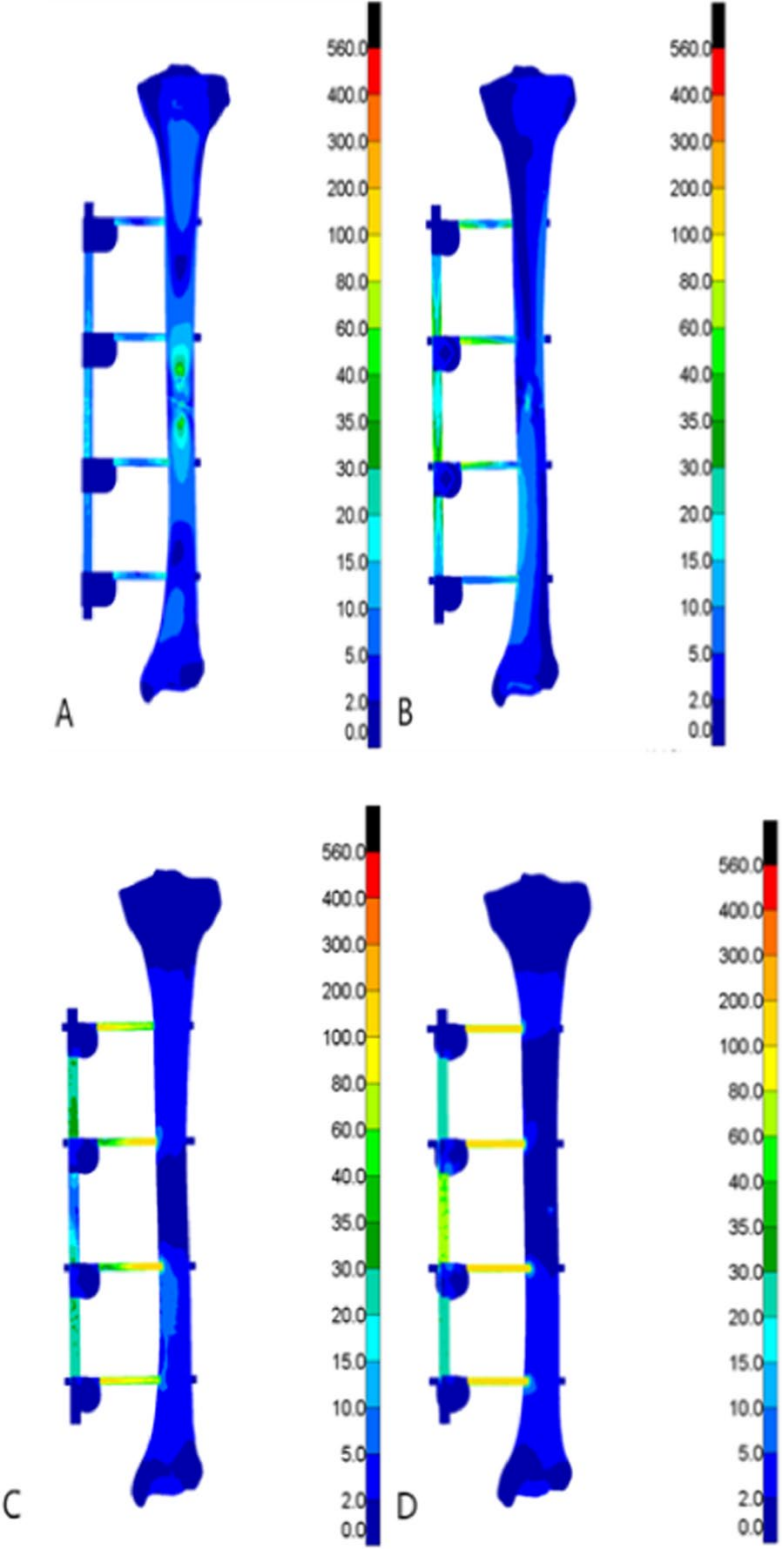
Von Mises stress clouds under four experiments in the external fixation bracket group (MPa). **A** Four-point bending experiment; **B** axial compression experiment; **C** clockwise torsion experiment; D counterclockwise torsion experiment

**Fig. 5:**
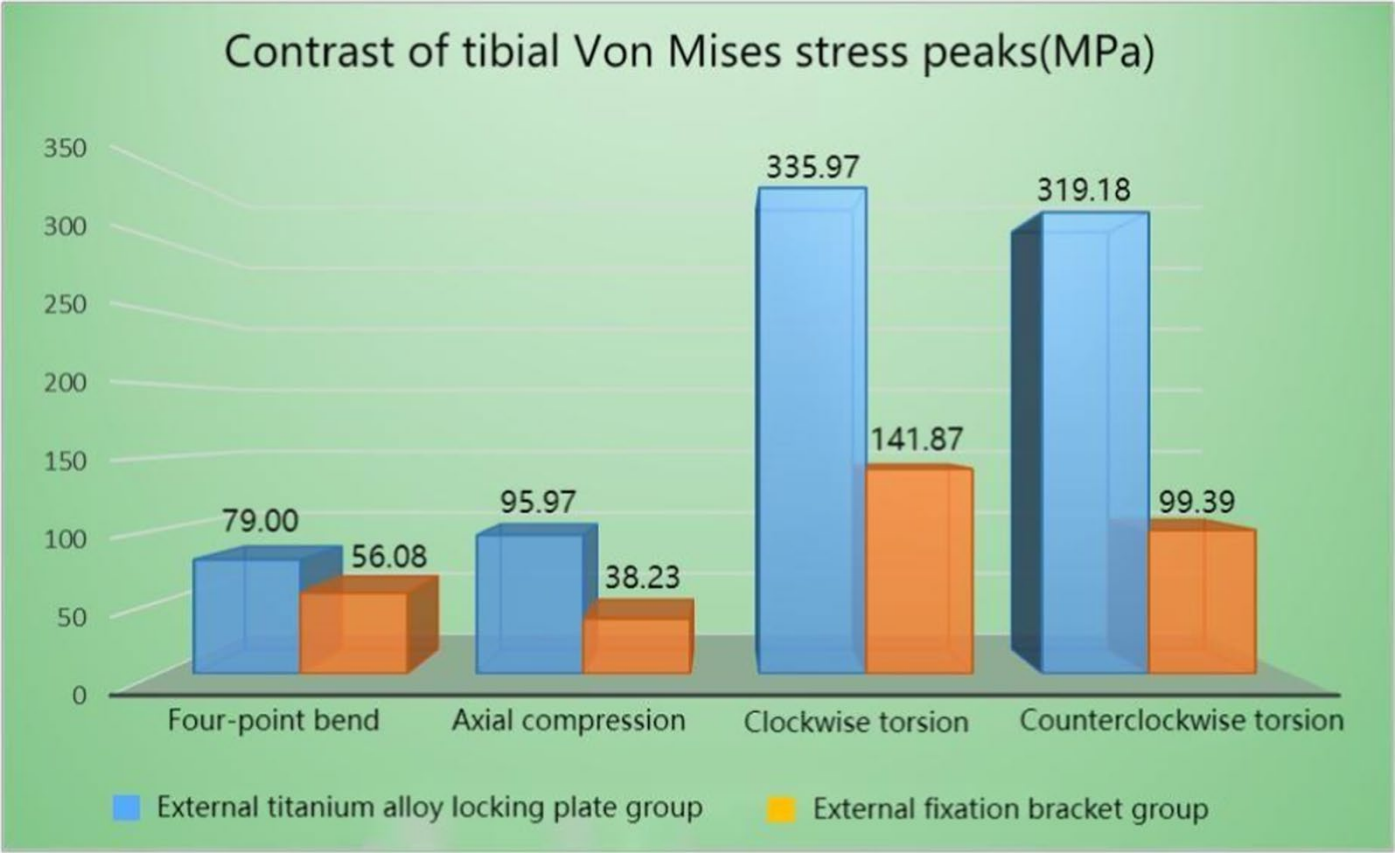
Von Mises stress peaks of the tibia under four experiments

**Fig. 6:**
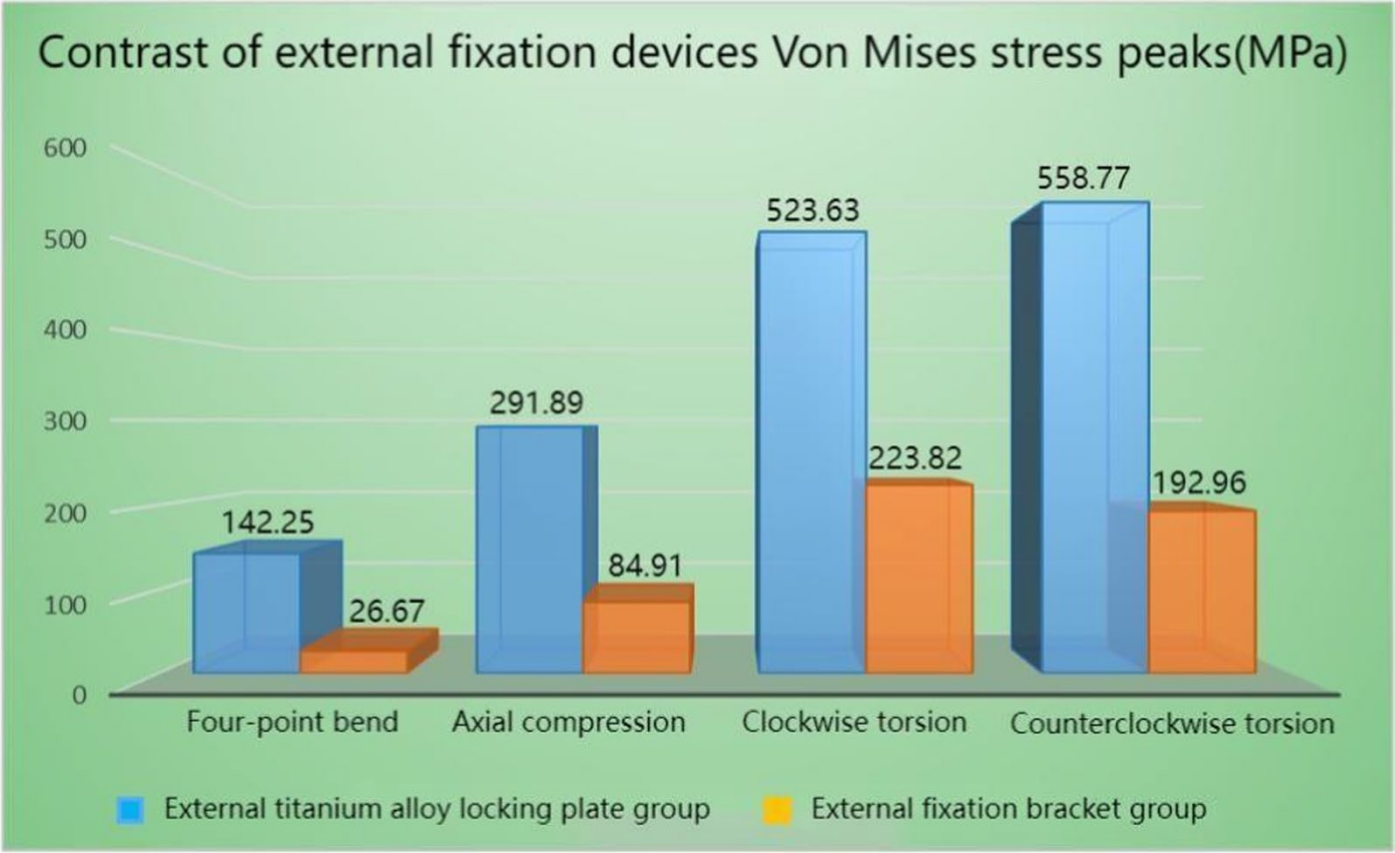
Von Mises stress peaks of external fixation devices under four experiments

In all the four test conditions, the stress of the tibia under the maximum load was mainly distributed around the fracture end and the cortical bone at the upper and lower ends of the tibia after Type AO/OTA 42A2 fractures treated with both external fixation techniques. To be more specific, the stress of the external titanium alloy locking plate model was mainly distributed at the contacts between the screw and tibia, and between the screw and titanium alloy locking plate around the fracture end. The stress of the external fixation bracket model was mainly distributed at the contacts between the self-tapping self-drilling needle and the tibia, and between the self-tapping self-drilling needle and the bracket around the fracture end. The peak stress detected in the two models ranged from 26.67 to 558.77 MPa, and the peak stress of the external titanium alloy locking plate model was greater than that of the external fixation bracket model. For the external titanium alloy locking plate model, the peak stress of the tibia was observed in the clockwise rotation test (335.97 MPa), while that of the fixation structure was observed in the counterclockwise rotation test (558.77 MPa). For the external fixation bracket model, the peak stress of the tibia (141.87 MP) and the external fixation device (223.82 MPa) was both observed in the counterclockwise rotation test. The stress distribution cloud maps and the specific data of the two external fixation techniques are shown in Figs. 3, 4, 5, 6, Tables 3, and 4, respectively.

**Table 3:** Compared with the external fixation bracket group, the percentage of von Mises stress peaks increment of tibia in the external titanium alloy locking plate group (%)

	External titanium alloy locking plate group
Four-point bend	40.87
Axial compression	151.03
Clockwise torsion	136.82
Counterclockwise torsion	221.14

**Table 4:** Compared with the external fixation bracket group, the percentage of von Mises stress peaks increment of external fixation devices in the external titanium alloy locking plate group (%)

	External titanium alloy locking plate group
Four-point bend	433.37
Axial compression	243.76
Clockwise torsion	133.95
Counterclockwise torsion	189.58

### Overall tibial displacement

**Fig.7:**
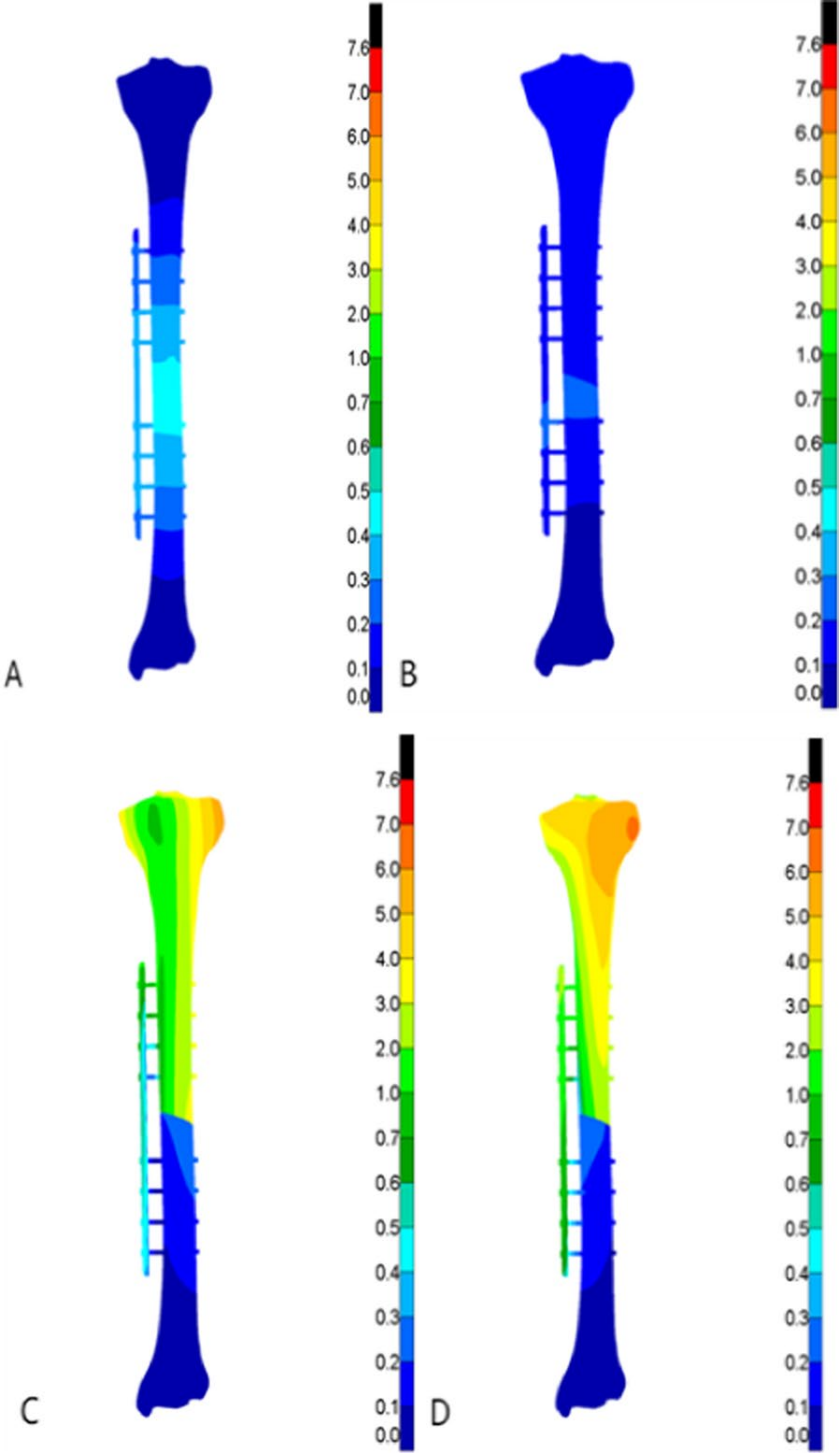
Displacement cloud diagram of external titanium alloy locking plate group structure (mm). **A** Four-point bending experiment; **B** axial compression experiment; **C** clockwise torsion experiment; D counterclockwise torsion experiment

**Fig. 8:**
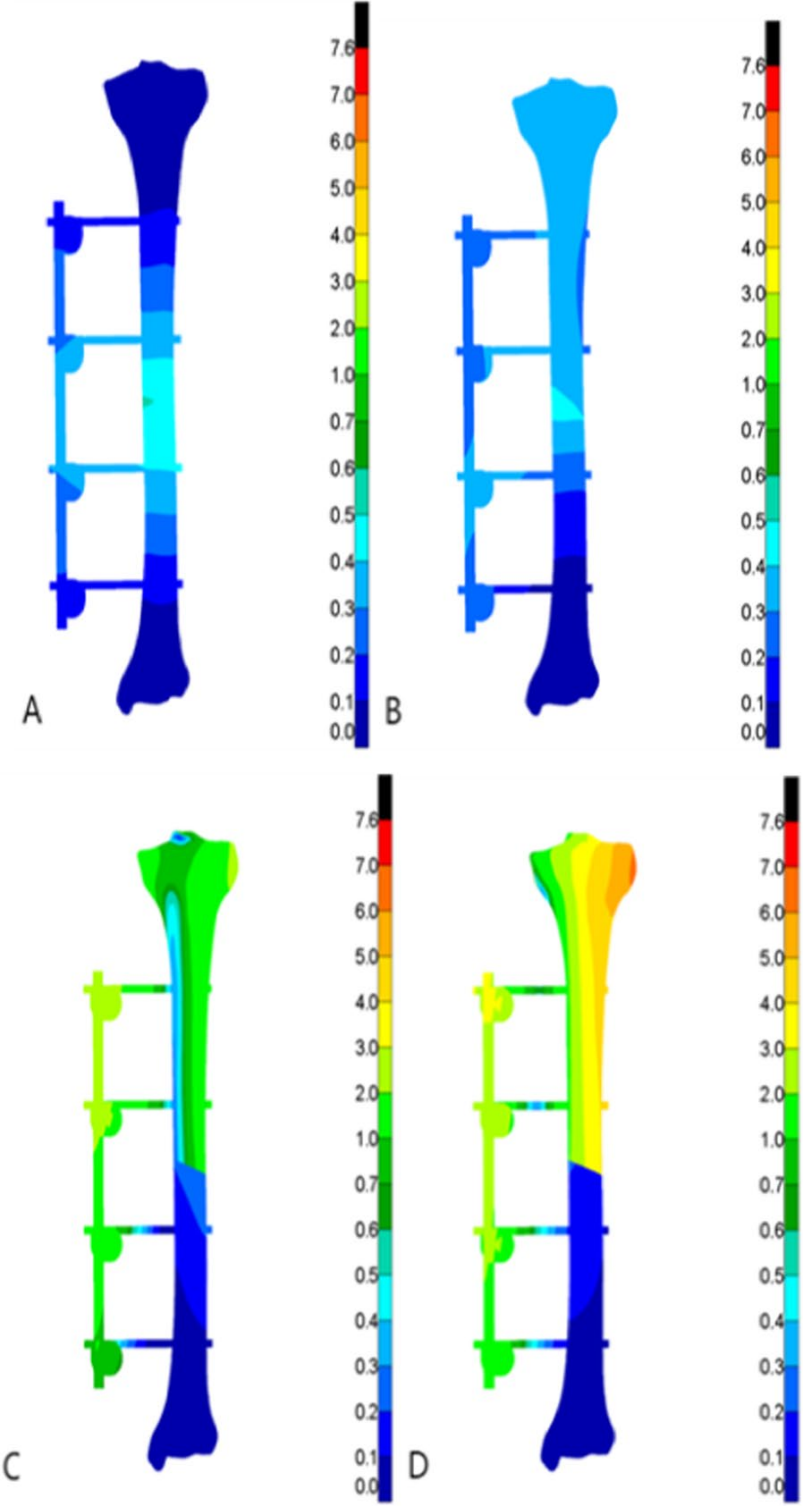
Displacement cloud diagram of the structure of the external fixation bracket group (mm). **A** Four-point bending experiment; **B** axial compression experiment; **C** clockwise torsion experiment; D counterclockwise torsion experiment

**Fig. 9:**
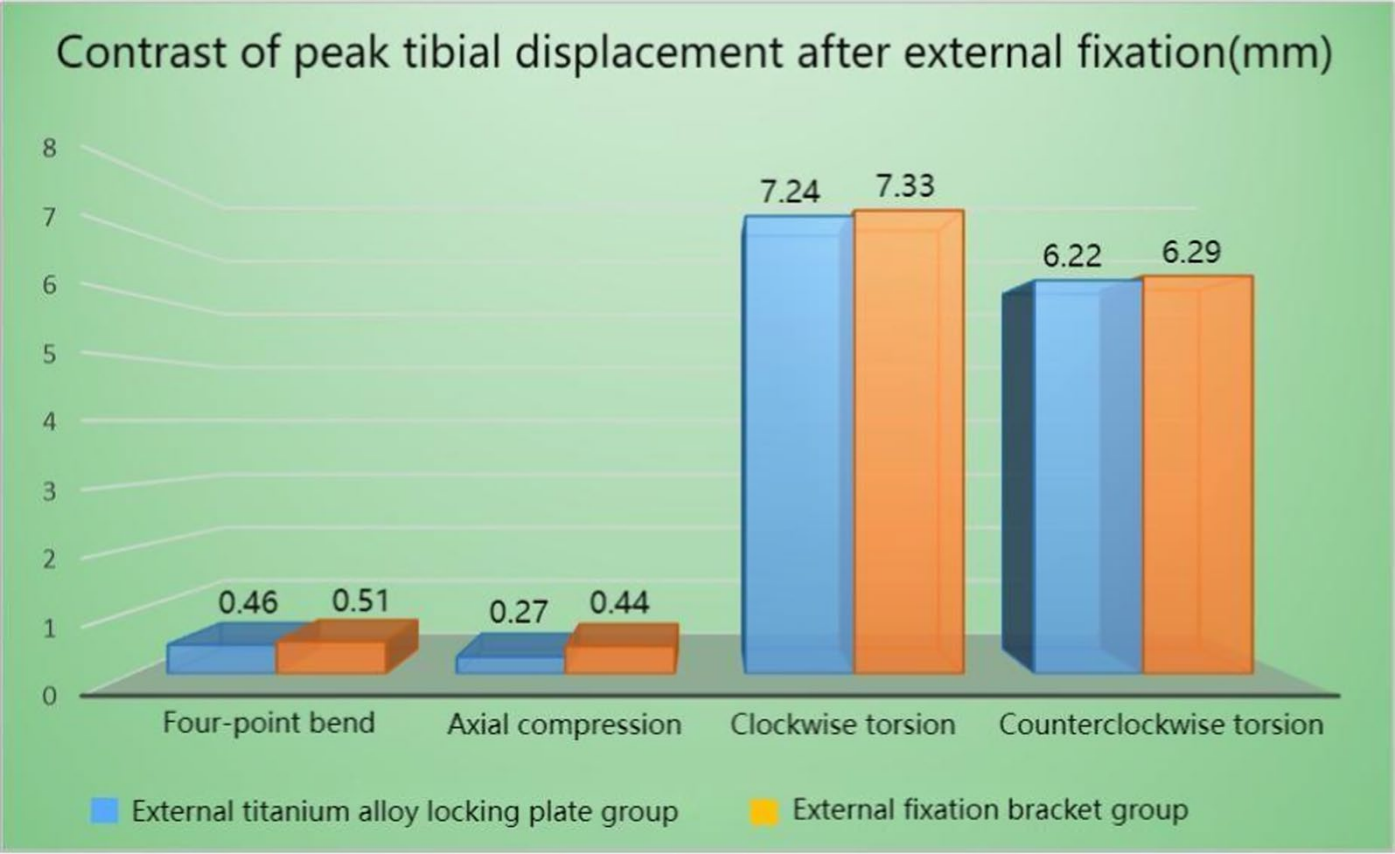
Comparison of the overall displacement of tibia in the two groups of external fixation methods under four experiments

In the four test conditions, the peak tibial displacement of the external titanium alloy locking plate model under the maximum load ranged from 0.27 to 7.24 mm, while that of the external fixation bracket model ranged from 0.44 to 7.33 mm. The most significant difference between the two techniques was observed in the axial compression test. Compared with the external titanium alloy locking plate model, the overall tibial displacement of the external fixation bracket model was larger by approximately 1.13–62.96%. The peak displacement cloud maps and the specific data of the two models are shown in Figs. 7, 8, 9, and Table 5, respectively.

**Table 5:** The increase in the overall displacement of the tibia in the external fixation frame group compared to the external titanium alloy locking plate group percentage (%)

	External fixation bracket group
Four-point bend	10.87
Axial compression	62.96
Clockwise torsion	1.24
Counterclockwise torsion	1.13

## Discussion

Due to a special anatomical structure and thin subcutaneous tissues, Type AO/OTA 42A2 fractures are often open fractures [26]. For the treatment of open tibial fractures, internal fixation surgeries can easily cause wound infection, skin and tissue necrosis, and even non-union of the fracture [27]. As a traditional external fixation method, fixation brackets have shown good effects in controlling severe open tibial fractures in clinical practice [28]. However, external fixation brackets are usually bulky and heavy, and have a considerable gap from the skin, often bringing inconvenience to the patients’ daily life. In contrast, recent clinical evidence indicates that external titanium alloy locking plates are characterized by advantages of small size, lightweight, easiness to operate, and low notching, and have shown better clinical efficacy in treating open fractures compared to traditional external fixation brackets [29–32]. Shon and Park [33] demonstrated that MIPO placement on the medial or lateral sides of the tibia could both achieve excellent results. Zhang et al. [34] reported that the anatomical locking plates were more advantageous when placed on the lateral side of the tibia. However, there are few reports of finite element biomechanical analysis on the external titanium alloy locking plates and external fixation brackets being placed on the medial side of the tibial fracture.

In this study, we carried out quantitative and qualitative biomechanical investigations on two different external fixation techniques for Type AO/OTA 42A2 fractures (i.e., external titanium alloy locking plates and external fixation brackets), and compared these two techniques in terms of stress conditions. On the basis of biomechanical principles, structural engineering concepts, treatment characteristics, and protocols in clinical practice, we conducted comprehensive 3D simulations on the biomechanical characteristics of the tibia and the external fixation devices for both techniques. Traditionally, the standard rod length of external fixation bracket for large bone is 11 mm, but in this study, the 8 mm protocol that is more widely used by clinicians was adopted, as it could provide better moment of inertia and stability and was expected to show better biological performance, thereby conducive to improving the robustness of the comparison results against the titanium alloy locking plate technique. Our results indicate that, in the finite element simulations of the four test conditions (four-point bending, axial compression, clockwise rotation, and counterclockwise rotation) under the maximum load, the peak stress of the external fixation devices of both techniques was ranged 26.67–558.77 MPa, all below the yield stress strength of titanium alloy (795 MPa) [35]. Basically, no fracture failure occurred, and both external fixation devices can meet the strength requirements.

More specifically, the peak stress of the external fixation device of the external titanium alloy locking plate model was mostly found at the contact between the screw and the bone plate around the fracture end, while that of the external fixation bracket model was mostly found at the contact between the self-tapping self-drilling needle and the bone cortex around the fracture end. This is mainly because the stability of the titanium alloy locking plate during fracture fixation is mainly provided by the special angular structure formed between the titanium alloy plate and the screw, while the stability of external fixation bracket mostly relies on the friction force exerted by the bone plate on the bone surface [36].

In the external titanium alloy locking plate model, more stress distribution was absorbed by the external fixation device, which is conducive to avoiding excessive stress distribution on the tibia so as to protect the periosteum from unnecessary injury, stimulate bone growth, and reduce the possibility of delayed union or even non-union of the fracture [37]. However, it also raises higher material requirements on the titanium alloy plate. For the stress distribution on the tibia, the peak stress was mainly detected near the intersections between external fixation devices and the bone, near the bone fracture ends, and near the cortical bones at the upper and lower ends of the tibia for both external fixation techniques. This indicates that both external fixation techniques can exert a compressive action on the fracture end, which is conducive to fracture healing. In comparison, the external titanium alloy locking plate technique can provide a stronger compressive effect on the fracture end while bearing greater stress with its external fixation device. The stress concentration on the cortical bones at the upper and lower ends of the tibia is considered to be caused by the anatomical structure of the tibia that connects between the knee joint and the ankle joint when simulating the stress condition of normal individuals. The stress concentration near the intersection between external fixation devices and the bone is considered to be caused by the interaction of the screw or screw rod with the tibia when subjected to stress under specified test conditions. As a matter of fact, the focus of this study is the stress concentration on the external fixation devices, rather than on the bone, as well as the overall displacement of the tibia. Although the peak stress of the tibia in the external titanium alloy locking plate group is greater than that in the external fixation bracket group under all concerned test conditions, an approximate comparison over the stress situation of the tibia between the two groups is still meaningful.

By comparing the displacement data of the two different external fixation techniques in four different test conditions (four-point bending, axial compression, clockwise rotation, and counterclockwise rotation), it was found that the peak tibial displacement of the external titanium alloy locking plate model under the maximum load was lower than that of the traditional external fixation bracket model, indicating that external titanium alloy locking plates have better compression performance in resisting axial force. In the four-point bending and clockwise/counterclockwise rotation tests, there were no significant differences in the peak tibial displacement between the two techniques, but that of external titanium alloy locking plate model was still generally lower than that of the external fixation bracket model. The results suggest that the external titanium alloy locking plate technique was also superior to the external fixation bracket technique in terms of torsion and bending force resistance. On the basis of the comparison of peak tibial displacement data across four different test conditions, it can be concluded that external titanium alloy locking plates were superior to unilateral external fixation brackets in terms of structural stability.

Limitations of this study should be highlighted. First, due to modeling difficulty, the effects of muscles, fascia, and soft tissues on the overall structure were not considered. Second, we only compared the two different external fixation techniques based on the most commonly-used protocol in clinical practice. As a matter of fact, the external fixation protocol may be customized depending on each patient’s individual conditions.

Although the results obtained from 3D finite element simulation cannot fully represent actual clinical scenarios, it can help us draw important biomechanical conclusions and provide mechanical guidance for the clinical research on external fixation surgeries after tibial fractures. Overall, the application of the finite element method has greatly reduced the dependence on the use of a large number of corpses or animal experiments in clinical practice, and can help illustrate the mechanical behavioral changes from a theoretical perspective. This method plays a positive role in promoting the development of biomechanics research for external fixation of tibial fractures.

## Conclusion

In summary, external titanium alloy locking plates are not only lightweight and easy-to-operate in fixing Type AO/OTA 42A2 fractures, but also performs better in resisting axial compression, bending force, and torsion. According to the results of finite element biomechanical analysis, the external titanium alloy locking plates technique is superior to the traditional external fixation bracket technique in fixing Type AO/OTA 42A2 fractures and can better meet the needs of clinical applications.

## Data Availability

The datasets used and/or analyzed during the current study are available from the corresponding author on reasonable request.

## Abbreviations

CT: Computed tomography
DICOM: Digital imaging and communications in medicine
STL: Stereolithography
STP (STEP): Standard for the exchange of product model data
BDF: Glyph bitmap distribution format
3D: Three-dimensional
FE: Finite element
VMS: Von Mises stress
